# sNASP Mutation Aggravates to the TLR4-Mediated Inflammation in SLE by TAK1 Pathway

**DOI:** 10.1155/2023/4877700

**Published:** 2023-09-20

**Authors:** Yatao Bao, Meng Lian, Yong Chen, Xiaotian Gu, Kunyu Cao, Xiaoping Du, Jiyu Ju

**Affiliations:** ^1^School of Basic Medical Science, Weifang Medical University, Weifang 261053, China; ^2^Medical Control Office, The Second Affiliated Hospital of Weifang Medical University, Weifang 261041, China; ^3^Medical Control Office, Weifang, No. 2 Hospital, Weifang 261041, China

## Abstract

Genetic factors play an important role in the pathogenesis of systemic lupus erythematosus (SLE), and abnormal Toll-like receptor (TLR) signaling pathways are closely related to the onset of SLE. In previous studies, we found that the mutant somatic nuclear autoantigenic sperm protein (sNASP) gene in the mouse lupus susceptibility locus *Sle2* can promote the development of lupus model mice, but the mechanism is still unclear. Here, we stimulated mouse peritoneal macrophages with different concentrations of lipopolysaccharide. The results showed that sNASP gene mutations can promote the response of the TLR4–TAK1 signaling pathway but have no significant effect on the TLR4–TBK1 signaling pathway. sNASP mutations enhanced TLR4-mediated nuclear factor-*κ*-gene binding and mitogen-activated protein kinase activation and IL-6, tumor necrosis factor secretion in murine peritoneal macrophages. Collectively, our study revealed the impact of sNASP gene mutation on the sensitivity of TLR4 receptors in mouse peritoneal macrophages and shed light on potential mechanisms underlying inflammation in autoimmune diseases.

## 1. Introduction

Systemic lupus erythematosus (SLE) is a multifactorial autoimmune disease characterized by the production of autoantibodies and subsequent inflammation that affects multiple organs [1, 2]. The etiology of SLE involves a complex interplay between genetic and environmental factors. While genome-wide association studies have successfully identified more than 100 susceptibility gene loci associated with SLE [3], the specific genes responsible for its clinical pathogenicity remain to be elucidated.

Mouse models have significantly contributed to our understanding of the pathogenesis of SLE [4]. In the NZM2410 model, three susceptibility loci associated with lupus, namely Sle1, Sle2, and Sle3, have been identified [5]. In our previous study, we utilized whole exome sequencing to identify a variant of somatic nuclear autoantigenic sperm protein (sNASP) from the Sle2 locus. This variant harbored two mutations in exon 10, specifically 841G>A and 844C>T in the sNASP cDNA sequence, resulting in the substitution of two consecutive amino acid residues (V281I and L282F) within the sNASP protein. Based on these findings, we hypothesized that the mutated sNASP represents a pathogenic gene at the Sle2 locus. To validate this hypothesis, we introduced the mutated sNASP into B6.lpr mice, generating B6.∆sNASP.lpr mice. In comparison to the control B6.lpr mice, the B6.∆sNASP.lpr mice exhibited enlarged spleen and lymph nodes, elevated total counts of T and B cells, increased activation and effector T cells, heightened levels of autoantibodies, and significantly enhanced inflammation in the kidneys and lungs [6]. Furthermore, we introduced the sNASP mutation into Sle1.Yaa mice, which demonstrated higher proportions of CD3^+^ T cells and activated CD19^+^CD86^+^ B cells in the spleen and lymph nodes compared to Sle1.Yaa mice [7].

Numerous studies have highlighted the role of sNASP, a histone chaperone protein, in the storage and transportation of histones, particularly histones H1, H3, and H4. sNASP is involved in regulating the dynamic balance of nucleosome formation, chromatin assembly, and disassembly, thereby influencing the conformation and accessibility of chromatin [8, 9]. Importantly, sNASP has the ability to bind to TRAF6 in unstimulated macrophages and prevent its own ubiquitination and subsequent degradation, thus exerting a negative regulatory effect on the Toll-like receptor (TLR) signaling pathway [10]. Recent studies have observed elevated mRNA expression levels of TLR4, TLR7, and TLR9 in SLE patients compared to healthy individuals [11, 12]. In vitro experiments have shown that mutations in TLR7 lead to increased production of inflammatory factors and elevated autoantibody titers [13], suggesting a crucial role for TLRs in the pathogenesis of SLE.

The impact of sNASP gene mutation on the TLR signaling pathway remains poorly understood. In this study, we employed various concentrations of lipopolysaccharide (LPS) to stimulate mouse peritoneal macrophages, aiming to elucidate the effects of sNASP gene mutations on the TLR4 signaling pathway and the subsequent production of inflammatory cytokines. Our research findings are expected to offer novel insights into the mechanistic role of sNASP mutation in promoting SLE in mice.

## 2. Material and Methods

### 2.1. Mice

Cyagen Biosciences Inc. (Guangzhou, Guangdong, China) generated the B6.∆sNASP mouse by introducing mutated bases of the sNASP allele into the C57BL/6J (B6) genome [6]. Briefly, two homology arms were made through PCR using BAC clones RP24-384F21 and RP24-72F14 from the B6 library as a template. The CTGTACTCCATGAGC sequence in exon 10 of the sNASP gene was edited to CTATATTCCATGAGC in the 5′ homology arm. The constructed targeting vector was electroporated into embryonic stem (ES) cells of C57BL/6 mouse, and then selected positive ES clones were microinjected into blastocysts. After genotyping, chimeric mice were bred with an Flp-deleter mouse to create an F1 mouse through Flp-mediated recombination. Finally, F1 mice intercrossed to obtain a homozygous B6.∆sNASP model. The B6.∆sNASP.lpr line was derived by breeding male B6.∆sNASP mice-to-female B6.lpr mice and subsequent intercrossing of progeny. B6.lpr was purchased from the Jackson Laboratory (Bar Harbor, ME, USA). B6.WT (C57BL/6) mice were provided by the Experimental Animal Center of Weifang Medical University. Female mice aged 3 months were used in this experiment. All animals were cared for under experimental protocols approved by the Weifang Medical University Animal Care Committee and housed in a specific pathogen-free facility.

### 2.2. Reagents and Abs

LPS (*Escherichia coli*, 0111: B4) was from Sigma-Aldrich (St. Louis, MO). The final concentrations of agonists were used as follows: 100, 10, 1 ng/ml. The Abs specific to anti–p-TAK1 (Ser439) (catalog number: P01458), anti-MAP3K7 (catalog number: BM5328), and anti-NAK/TBK1 (catalog number: A00261-1) were from Boster Technology (Wuhan, China); anti-p-JNK (catalog number: AF1762), anti-JNK (catalog number: AF1048), anti-p-p38 (catalog number: AF5887), anti-p38 (catalog number: AF1111), anti-IKB-*α* (catalog number: AF1282), anti-IRF3 (catalog number: AF2485) and an horseradish peroxidase-conjugated secondary Abs (catalog number: A0208) were from Beyotime Technology (Shanghai, China). Anti-TBK1/NAK (Ser172) (catalog number: #5483) and p-IKB-*α* (Ser32) (catalog number: #2859) were from Cell Signaling Technology (Beverly, MA). Anti-p-IRF3 (catalog number: bs-9278R) was from Bioss Technology. Anti-NASP (sc-398379) and anti-TRAF6 (sc-8409) were from Santa Cruz Biotechnology (Santa Cruz, CA).

### 2.3. Acquisition of Peritoneal Macrophages and LPS Stimulation

To obtain mouse primary peritoneal macrophages, a mouse was euthanized using carbon dioxide and sprayed with 75% ethanol. Scissors were used to expose the inner skin lining the peritoneal cavity, following which peritoneal lavage was performed using phosphate buffer saline with 3% fetal calf serum. Samples were then centrifuged and reconstituted into fresh culture media. The cells were next transferred to a 6-well plate with the number of 1 × 10^6^ cells per well and incubated for 3 hr. After incubation, the media was changed to remove free-floating cells. Three hours later, nonadherent cells were removed, and the adherent monolayer cells were used as peritoneal macrophages [14]. The cells were cultured at 37°C under 5% CO_2_ in RPMI 1640 supplemented with 12% fetal bovine serum. After peritoneal macrophages were stimulated with LPS (1, 10, 100, 0/0.5/1/2 hr), the supernatants of cell culture were collected for the detection of cytokines, and the cell precipitates were collected for quantitative polymerase chain reaction (qPCR) and Western blot detection.

### 2.4. ELISA

The peritoneal macrophages were stimulated with LPS (1, 10, 100 ng/ml, 0/0.5/1/2 hr), and cell culture supernatants were assayed for IL-6 (catalog number: 88-7064), TNF-*α* (catalog number: 88-7324) and IFN-*α* (catalog number: BMS6027) by enzyme-linked immunosorbent assay (ELISA) kits (Invitrogen Biotech, USA) following the manufacturer's directions.

### 2.5. Western Blot

For immunoblot analysis, cells were lysed with Servicebio (Wuhan, China) RIPA Lysis Buffer (catalog number: G2002) supplemented with a protease inhibitor mixture (catalog number: G2007; Servicebio), and then the protein concentrations of the intermixture were measured with a bicinchoninic acid assay (catalog number: P0012; Beyotime). Equal amounts of extracts were separated by SDS–PAGE and then were transferred onto polyvinylidene fluoride membranes for immunoblot analysis.

### 2.6. Real-Time Reverse Transcription-Polymerase Chain Reaction

RNA was extracted from cells using the RNAex pro reagent (code no: AG21101-S, Accurate Biotechnology, Hunan, China) and converted into cDNA by reverse transcription using the Evo M-MLV RT Premix Kit (code no: AG11706, Accurate Biotechnology, Hunan, China). The sequences of primers were (forward) 5′-CAGGCGGTGCCTATGTCTC-3′ and (reverse) 5′-CGATCACCCCGAAGTTCAGTAG-3′ for TNF-*α*, (forward) 5′-TGTGCAATGGGCAATTCTGAT-3′ and (reverse) 5′-GGTACTCCAGAAGACCAGAGGA-3′ for IL-6 and (forward) 5′-TCTTTGCAGCTCCTTCGTTGCCGGTCC-3′ and (reverse) 5′-GTCCTTCTGACCCATTCCCACCATCCAC-3′ for *β*-actin.

### 2.7. Statistical Analysis

Results are expressed as means ± SEM. Data were analyzed using an independent samples *t*-test using origin 2021 and SPSS 26 software. A *p*-value < 0.05 was considered significant error bars depicting structural equation modeling. 0.05

## 3. Result

### 3.1. sNASP Mutation Promotes TLR4-Induced Inflammatory Cytokines Expression

**Figure 1:**
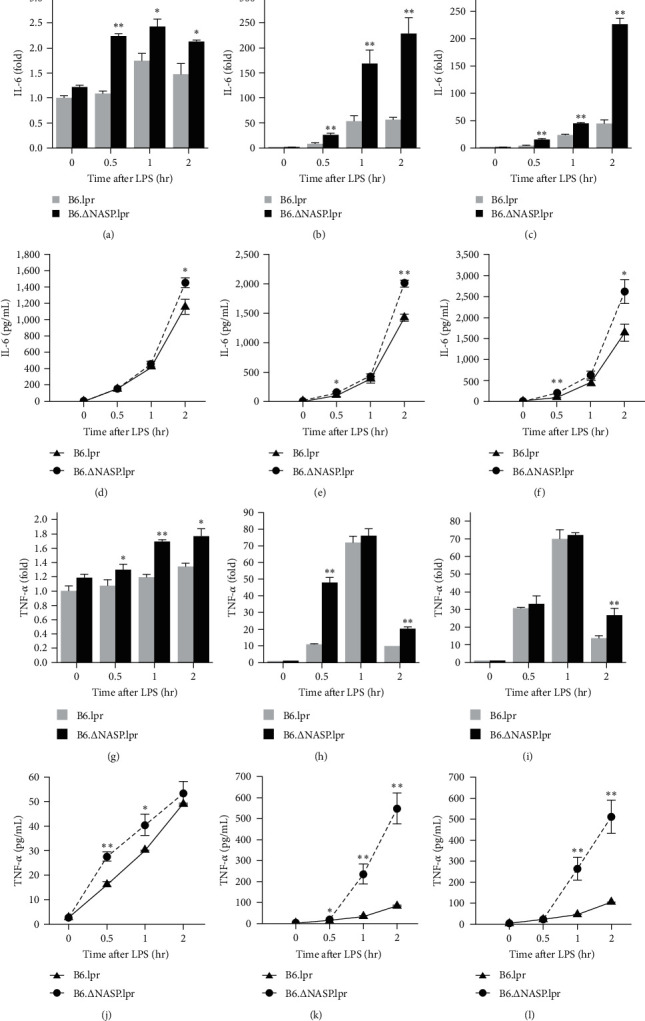
*sNASP* mutation promotes LPS-induced proinflammatory cytokine production in B6.lpr mice. The expression of mRNA ((a)–(c) and (g)–(i)) and protein ((d)–(f) and (j)–(k)) of IL-6 and TNF-*α* were measured by qPCR and ELISA in peritoneal macrophages, compared between B6.lpr and B6.∆sNASP.lpr mice. Following 0/0.5/1/2 hr stimulation with ((a), (d), (g), (j)) LPS 1 ng/ml, ((b), (e), (h), (k)) LPS 10 ng/ml, and ((c), (f), (i), (l)) LPS 100 ng/ml. Data were shown as means ± SEM (*n* = 3) of one representative experiment.  ^*∗*^*P* < 0.05,  ^*∗∗*^*P* < 0.01.

**Figure 2:**
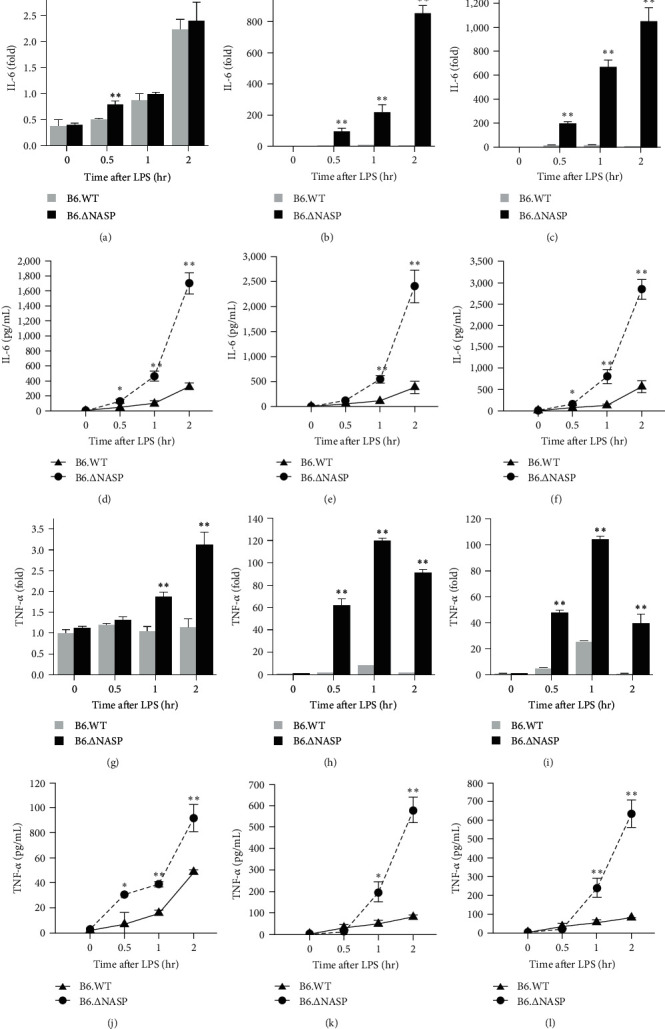
*sNASP* mutation augments LPS-induced the cytokine IL-6 and TNF-*α* expression in B6.WT mice. The peritoneal macrophages were treated with LPS for 0.5, 1, and 2 hr that IL-6 and TNF-*α* levels were analyzed by qPCR ((a)–(c), (g)–(i)) and ELISA ((d)–(f), (j)–(l)), compared between B6.WT and B6.∆sNASP mice. Following stimulation with ((a), (d), (g), (j)) LPS 1 ng/ml, ((b), (e), (h), (k)) LPS 10 ng/ml, and ((c), (f), (i), (l)) LPS 100 ng/ml. Data were shown as means ± SEM (*n* = 3) of one representative experiment.  ^*∗*^*P* < 0.05,  ^*∗∗*^*P* < 0.01.

The activation of the TLR4 signaling pathway ultimately leads to the release of inflammatory cytokines [15, 16]. Excessive expression of inflammatory factors exacerbates the inflammatory response [17]. In order to examine the potential inhibitory function of sNASP and whether the mutation of the sNASP gene could enhance the activation of the TLR4 receptor, resulting in the expression of inflammatory cytokines, we assessed the levels of IL-6 and TNF-*α* proteins and mRNAs in mouse peritoneal macrophages after stimulation with different concentrations of LPS using ELISA and qPCR. Following various levels of LPS stimulation, both the mRNA and protein levels of IL-6 in peritoneal macrophages from B6.∆sNASP.lpr mice were higher compared to those from B6.lpr mice (Figure 1(a)–1(f)). Similarly, the levels of TNF-*α* mRNA and protein were also increased (Figure 1(g)–1(l)). Additionally, the expression levels of IL-6 and TNF-*α* mRNAs and proteins in peritoneal macrophages from B6.∆sNASP mice were significantly higher than those from B6.WT mice following different concentrations of LPS stimulation (Figure 2(a)–2(l)). Taken together, these findings suggest that mutations in the sNASP gene can impact the sensitivity of the TLR4 receptor, leading to increased expression levels of IL-6 and TNF-*α* genes and proteins in mouse peritoneal macrophages.

### 3.2. sNASP Mutation Downregulates Protein Expression of TRAF6

**Figure 3:**
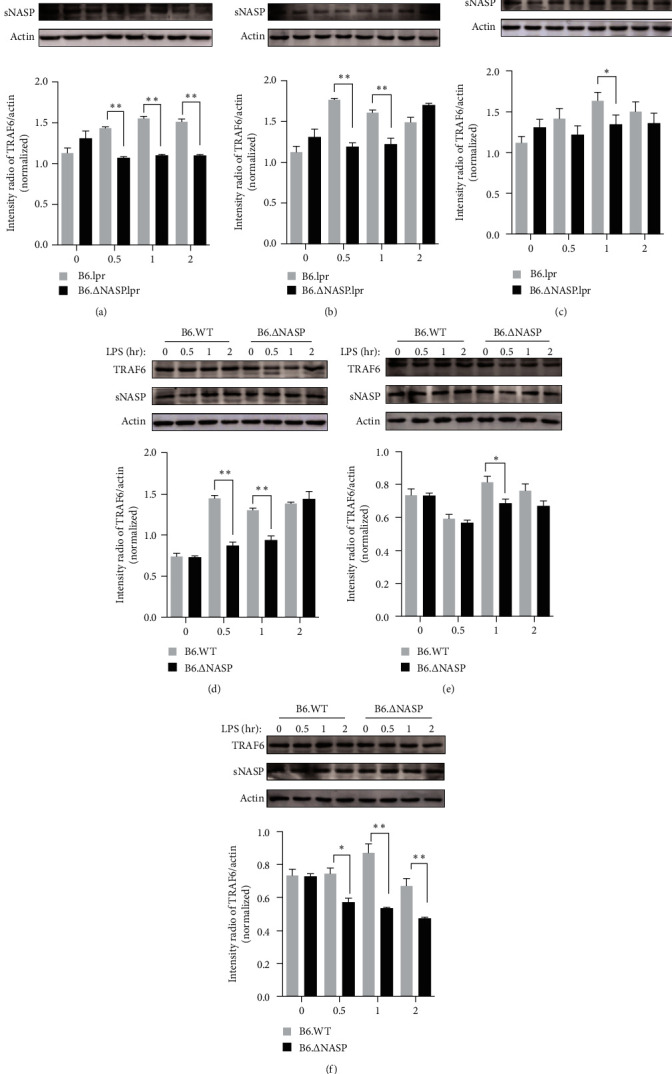
*sNASP* mutation downregulates the protein of TRAF6 expression. The expression of TRAF6 was measured by western blot in peritoneal macrophages and compared among B6.lpr, B6.∆sNASP.lpr, B6.WT, and B6.∆sNASP mice, and mouse *β*-actin was used as control. Following 0/0.5/1/2 hr stimulation with ((a) and (d)) LPS (1 ng/ml), ((b) and (e)) LPS (10 ng/ml), ((c) and (f)) LPS (100 ng/ml). Data were shown as means ± SEM.  ^*∗*^*P* < 0.05,  ^*∗∗*^*P* < 0.01; independent sample *t*-test. Results shown are representative of 2–3 independent experiments.

LPS, as a specific agonist for TLR4, can effectively activates sNASP, leading to its dissociation from the TRAF6 complex and subsequent ubiquitination and degradation of TRAF6. This activation triggers downstream signaling pathways and induces the release of inflammatory factors from the cells [10, 18]. To assess the expression of sNASP and TRAF6, we initially stimulated peritoneal macrophages obtained from B6.lpr mice, B6.∆NASP.lpr mice, B6.WT mice, and B6.∆NASP mice with different concentrations of LPS (1, 10, 100 ng/ml) and quantified the levels of sNASP and TRAF6 proteins. Interestingly, we did not observe any significant differences in the protein levels of sNASP in peritoneal macrophages among B6.∆NASP.lpr, B6.lpr, and B6.∆NASP mice compared to the B6.WT control (Figure 3(a)–3(f)). However, the expression of TRAF6 following LPS stimulation showed a significant reduction. This reduction in TRAF6 expression may indicate a change in the sensitivity of the TLR4 receptor, particularly evident at 1 hr post-LPS stimulation. These findings suggest that the sNASP mutation does not affect the quantity of sNASP but may influence its function, potentially contributing to TRAF6 degradation and subsequent activation of the downstream signaling pathway.

### 3.3. sNASP Mutation Promotes TLR4-Induced NF-*κ*B and MAPK Activation

**Figure 4:**
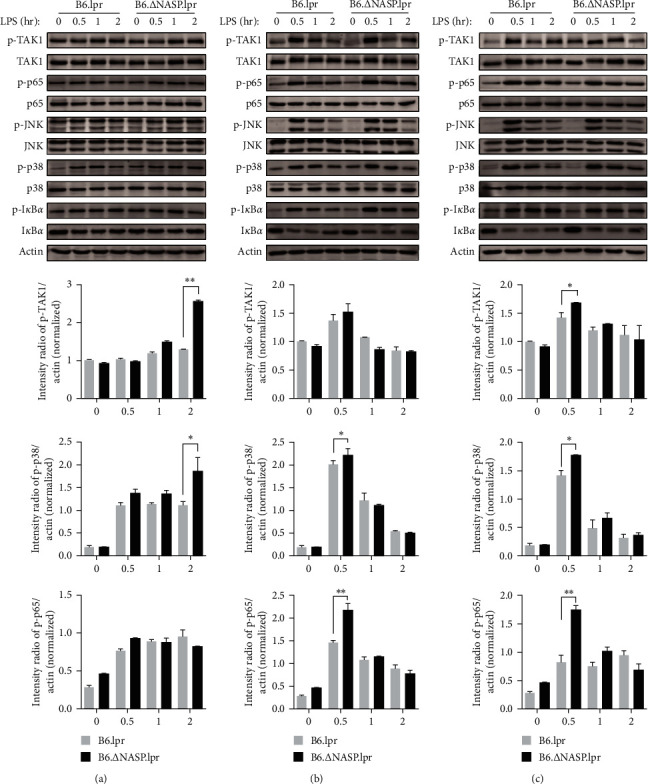
*sNASP* mutation promotes TLR4-induced NF-*κ*B and MAPK activation of the pathways in B6.lpr mice. The expression of phosphorylated and total proteins was measured by western blotting in peritoneal macrophages and compared between B6.lpr and B6.∆sNASP.lpr mice, and mouse *β*-actin was used as control. Following 0/0.5/1/2 hr stimulation with (a) LPS (1 ng/ml), (b) LPS (10 ng/ml), (c) LPS (100 ng/ml). Data were shown as means ± SEM.  ^*∗*^*P* < 0.05,  ^*∗∗*^*P* < 0.01; independent sample *t*-test. Results shown are representative of 2–3 independent experiments.

The activation of TLR4 involves two downstream signaling pathways, namely the TLR4–TAK1 pathway and the TLR4–TBK1 pathway. Here, mouse models (B6.lpr, B6.∆NASP.lpr, B6.WT, and B6.∆NASP) and western blot were used to investigate the protein dynamics alterations involved in TLR4–TAK1 pathway that is potentially under the modulation by TRAF6. Mouse peritoneal macrophages were stimulated with 1 ng/ml LPS for durations of 0.5, 1, and 2 hr. The levels of p-TAK1 and p-p38 in peritoneal macrophages of B6.∆NASP.lpr mice were higher than those in B6.lpr mice only after 2 hr of stimulation (Figure 4(a)). In the groups stimulated with 10 ng/ml and 100 ng/ml LPS, the levels of p-TAK1, p-p65, and p-p38 in peritoneal macrophages of B6.∆NASP.lpr mice were significantly higher than those in B6.lpr mice at 0.5 hr after LPS stimulation. There was no significant change in p-JNK protein (Figures 4(b) and 4(c)). These findings indicate that mutations in the sNASP gene increase the protein expression levels of the TLR4–TAK1 signaling pathway in mouse peritoneal macrophages compared to B6.lpr mice. In summary, the mutation in the sNASP gene enhances the sensitivity of the TLR4 receptor in B6.lpr mice, resulting in the activation of nuclear factor-*κ*-gene binding (NF-*κ*B) and mitogen-activated protein kinase (MAPK) pathways.

**Figure 5:**
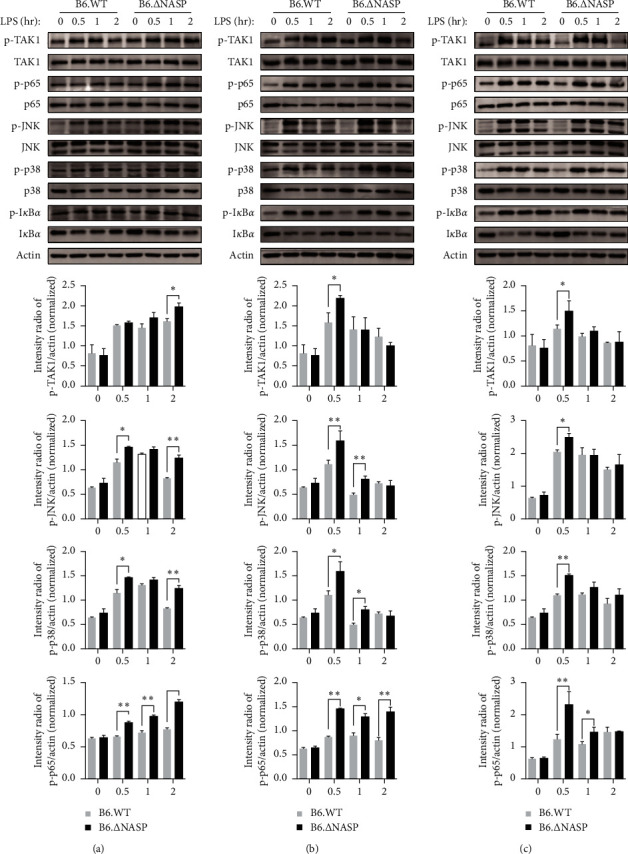
*sNASP* mutation accelerates the signaling of TLR4–TAK1 pathway in B6.WT mice. The phosphorylated and total protein levels were determined by immunoblotting in peritoneal macrophages following 0/0.5/1/2 hr stimulation with (a) LPS (1 ng/ml), (b) LPS (10 ng/ml), (c) LPS (100 ng/ml). Mouse *β*-actin was detected as control. Data were shown as means ± SEM.  ^*∗*^*P* < 0.05,  ^*∗∗*^*P* < 0.01; independent sample *t*-test. Results shown are representative of 2–3 independent experiments.

As Fas ligands have been shown to promote a TLR-dependent model of lupus-like inflammation [19], we sought to rule out the effect of Fas deficiency on TLR4–TAK1 pathway. The expression of p-TAK1, p-p65, p-JNK, p-p38, and p-I*κ*B*α* in peritoneal macrophages from both B6.WT mice and B6.∆NASP mice following stimulation with different concentrations of LPS under identical conditions were examined to test the hypothetical effects of Fas deficiency. After being stimulated with 1 ng/ml LPS for 2 hr, the expression levels of p-TAK1, p-p65, p-JNK, and p-p38 proteins in peritoneal macrophages from B6.∆NASP mice were higher compared to those from B6.WT mice (Figure 5(a)). Furthermore, the levels of p-TAK1, p-p65, and p-p38 in peritoneal macrophages from B6.∆NASP mice were significantly higher than those from B6.WT mice at 0.5 hr following stimulation with 10 and 100 ng/ml LPS (Figures 5(b) and 5(c)). Additionally, the expression level of p-JNK protein was also higher in B6.∆NASP mice compared to B6.WT mice at 0.5 hr after stimulation with 10 ng/ml LPS, but there was no difference observed at 100 ng/ml LPS. Overall, these observations suggest that mutations in the sNASP gene indeed lead to increased protein expression levels of the TLR4 TAK1 signaling pathway in mouse peritoneal macrophages, thereby enhancing the activation of the TLR4 receptor through the TLR4 TAK1 signaling pathway in response to LPS stimulation. Notably, a comparison between Figures 2 and 3 reveals distinct variations in p-p65 and p-TAK1 alterations at the lowest LPS concentration, implying an influence of the lpr mutation. In conclusion, the sNASP gene mutation in B6.WT mice genuinely improves the sensitivity of the TLR4 receptor compared to lpr mice, resulting in the activation of the NF-*κ*B and MAPK pathways independent of Fas.

### 3.4. sNASP Mutation Does Not Impact TLR4-Induced IRF3 Activation

**Figure 6:**
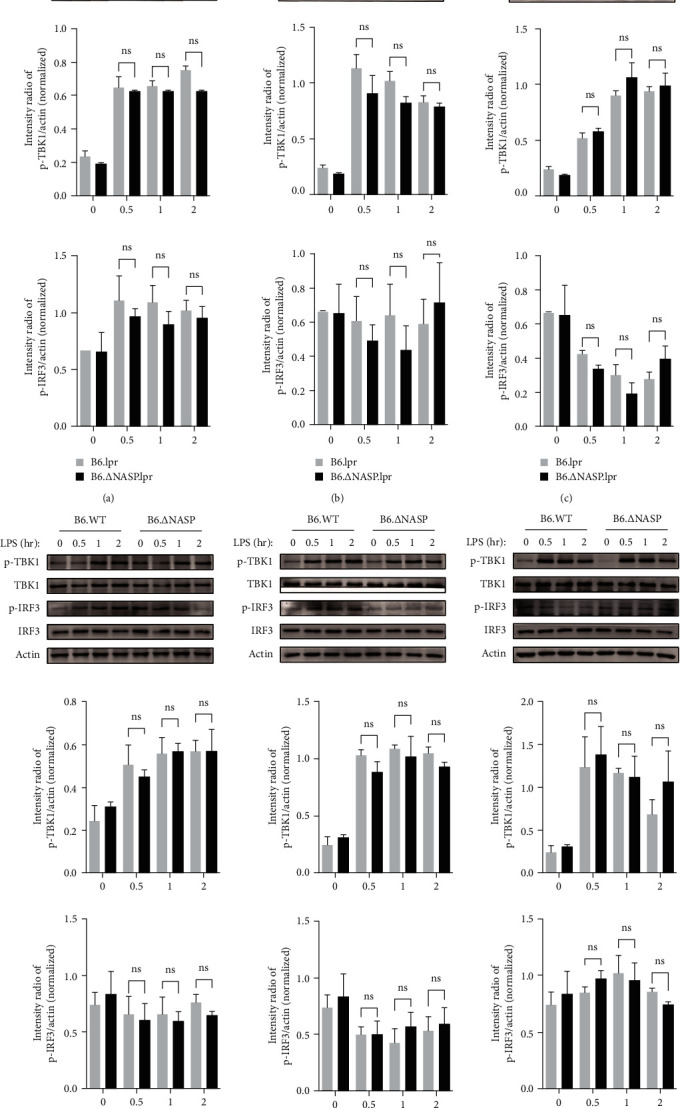
*sNASP* mutation has no influence on TLR4-induced IRF3 activation of the pathway. The expression of phosphorylated and total proteins were measured by western blotting in peritoneal macrophages and compared between ((a)–(c)) B6.lpr and B6.∆sNASP.lpr mice, ((d)–(f)) B6.WT and B6.∆sNASP mice, and mouse *β*-actin was used as an internal control. Following 0/0.5/1/2 hr stimulation with ((a) and (d)) LPS (1 ng/ml), ((b) and (e)) LPS (10 ng/ml), ((c) and (f)) LPS (100 ng/ml). Data were shown as means ± SEM.  ^*∗*^*P* < 0.05,  ^*∗∗*^*P* < 0.01; independent sample *t*-test. Results shown are representative of 2–3 independent experiments.

**Figure 7:**
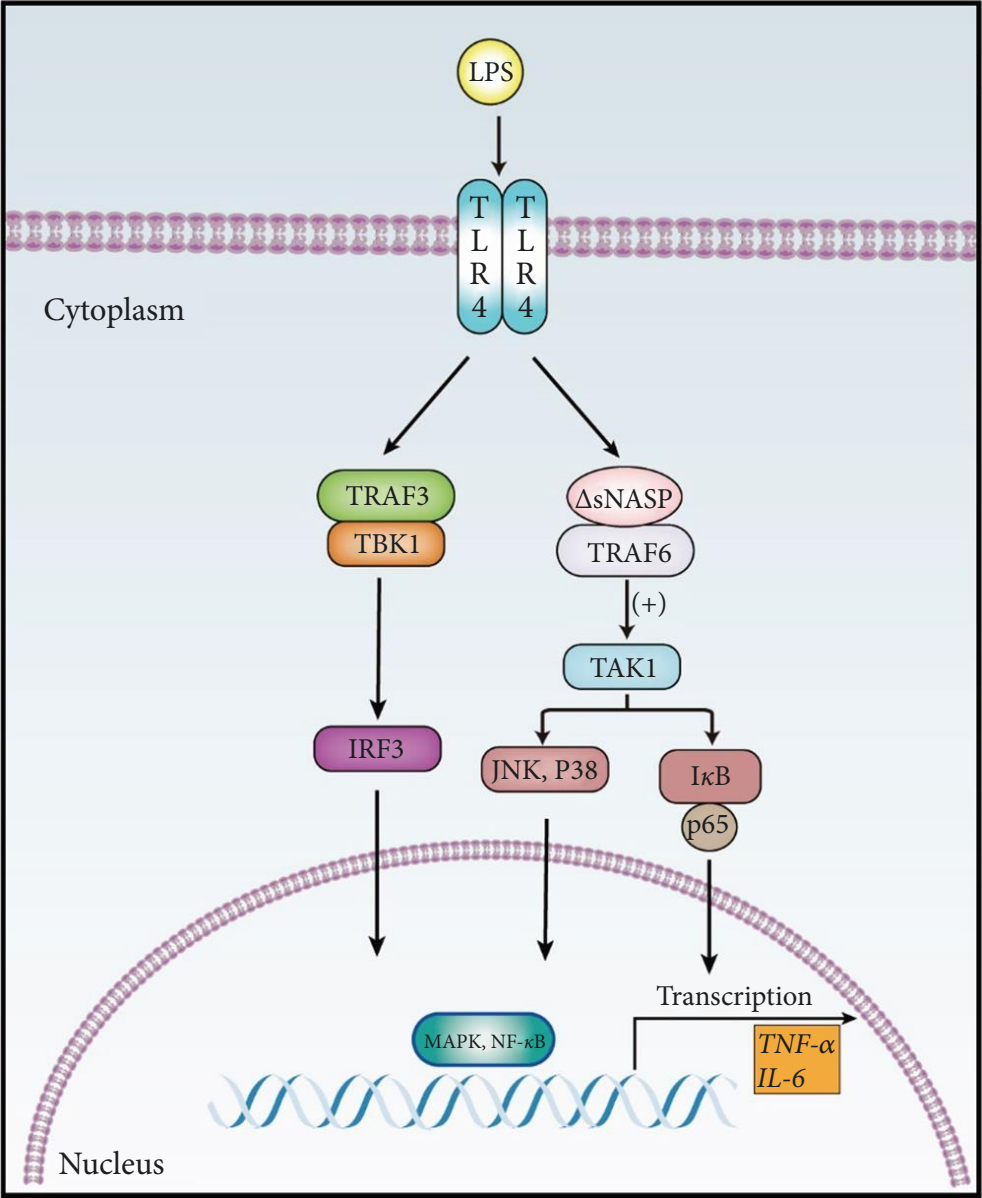
Model of TLR4 signaling pathway regulated by sNASP. *sNASP* mutation promotes LPS-induced TLR4–TAK1 activation in peritoneal macrophages and has no influence on TLR4–TBK1, resulting in the activation of NF-*κ*B and MAPK signaling pathways and the expression of proinflammatory cytokines.

To investigate the impact of mutation on the TLR4–TBK1 signaling pathway, we examined the protein expression levels of this pathway in mouse peritoneal macrophages after LPS stimulation using western blot analysis. Comparing B6.lpr mice with B6.∆sNASP.lpr, B6.WT, and B6.∆sNASP mice, the expression levels of TBK1 and phosphorylated IRF3 proteins in the TLR4–TBK1 signaling pathway did not show significant differences among the groups when stimulated with various concentrations of LPS (Figure 6(a)–6(f)). These findings indicate that there were no notable changes in protein levels within the TLR4–TBK1 signaling pathway following LPS stimulation between the B6.∆sNASP.lpr and B6.lpr groups, as well as the B6.∆sNASP and B6.WT groups. Additionally, type I interferons (IFN-*α*) are key cytokines involved in the innate immune response and are implicated in the pathogenesis of autoimmune diseases, such as SLE, mediated by the IRF3 pathway [20, 21]. IFN-*α* also did not exert influence in B6.lpr mice (Figure S1). Thus, different LPS stimulation did not significantly affect the sensitivity of the TLR4–TBK1 signaling pathway following sNASP gene mutation, suggesting that the activation of TLR4 by LPS does not drive the IRF3 signaling pathway. In conclusion, these results suggest that the mutation in sNASP may induce a proinflammatory response and enhance the activation of TLR4 through TAK1 pathway (Figure 7).

## 4. Discussion

The female NZB/WF1, MRL/lpr, and BXSB/Yaa mice are the most commonly used animal models for SLE due to their natural development of immunopathological symptoms that resemble those observed in SLE patients [22]. Obviously, lupus mouse model and SLE patients may have similar pathogenesis and some common genetic basis [23]. Therefore, finding and identifying the pathogenic genes of lupus mouse model will greatly help to clarify the etiology and pathogenesis of SLE. NZW mice were backcrossed with NZB/WF1 mice repeatedly to obtain an inbred strain of NZM2410 mice. Both male and female mice could spontaneously develop the same autoimmune diseases as lupus NZB/WF1 mice. In 1994, it was first reported that three lupus susceptibility loci associated with autoimmune diseases are called Sle1, Sle2, and Sle3, respectively. The Sle2 locus is located on chromosome 4, which has been found to regulate B-cell hyperactivity and expansion of B1a cells in NZM2410 mice that some gene mutations from the Sle2 locus, could promote the inflammatory response, such as Skint6 and sNASP [6, 24–26]. The variant of Skint6 W168X allele was confirmed as a pathogenic mutant gene that triggers autoimmune disease by producing a truncated Skint6 peptide binding the Skint6 receptors on T and B lymphocytes [26]. In addition, the variant of sNASP combined with the lpr mutation in the Fas gene enhances autoimmunity, resulting in more severe lupus nephritis and significantly increased lymphadenopathy in the B6.lpr strain [27].

Traditionally, sNASP is crucial in assembling chromosomes and is vital in the final stages of DNA replication and folding chromosomes. It binds to H1 and H4, participates in histone transport, and promotes cell proliferation [28, 29]. sNASP also maintains histone H3K9me1 to regulate chromatin accessibility [6]. Recently, another new biological function of protein sNASP has been discovered [10]. The cytoplasmic protein sNASP can bind to tumor necrosis factor receptor-associated factor 6 (TRAF6) and prevent the ubiquitination of TRAF6 and its downstream signal pathway and the production of inflammatory cytokines in unstimulated macrophages. However, after TLR4 was stimulated by LPS, sNASP was phosphorylated and separated from TRAF6. Then, TRAF6, which does not bind to sNASP protein, produces autologous ubiquitin, activating downstream signal pathways and initiating the production of inflammatory cytokines. In addition to TLR4, the stimulation of TLR1, TLR2, TLR5, and TLR6 can also mediate the phosphorylation of sNASP and its dissociation from TRAF6, so the protein sNASP can also negatively regulate these TLR signaling pathways and the production of inflammatory cytokines. TLR1, TLR2, TLR4, TLR5, and TLR6 are expressed on mononuclear macrophages, dendritic cells, and B cells, as well as on CD4^+^T and CD8^+^T cells [22]. It is suggested that the negative regulation of protein sNASP on TLR signaling pathway exists in innate immunity and adaptive immunity-related immune cells.

Aberrant activation of TLRs may disrupt immune homeostasis, which leads to excessive inflammatory cytokines aggravating the immune responses [30]. Although at present, the relationship between TLR and SLE is mostly focused on TLR7 and TLR9 because they are in the cytoplasm and can recognize nucleic acid antigens. Until recently, accumulating evidence shows that TLR4 also plays an important role in the pathogenesis of SLE [11, 31]. A penetration-conjugated small peptide (TIP3) could block cytokine production to ameliorate inflammatory response in mice models of arthritis and alleviate the disease symptoms of SLE models through the TLR4 pathway [32]. To test whether the mutation of sNASP gene alters the sensitivity of the TLR4 receptor, resulting in the difference between sNASP mutation groups and control groups in the activation level of TLR4 signaling, we measured the protein levels of the TLR4 signaling pathways and the quantified pro-inflammatory cytokine activations following different LPS stimulation in the peritoneal macrophages. As hypothesized, the mutation of sNASP produced excessive pro-inflammatory cytokines, specifically IL-6 and TNF-*α* at both mRNA and protein levels, which enhanced the activation of the TLR4–TAK1 signaling pathways.

Cytokine dysregulation aggravates immune dysfunction, leading to autoimmune diseases such as SLE [33]. The pro-inflammatory cytokines, which infer IL-6 as a mediator of disease initiation, increase the susceptivity of the vascular pathology in MRL-Fas^lpr^ mouse model [34]. A meta-analysis suggested that the levels of IL-6 and TNF-*α* in SLE patients were higher than in healthy controls [35]. Compared to B6.lpr, the proteins of IL-6 and TNF-*α* in macrophage culture supernatant in B6.∆sNASP.lpr mice were significantly higher, and the level of the mRNA was also increased by LPS stimulation. The same results were observed in B6.∆sNASP and B6.WT mice, suggesting that the mutation in sNASP gene increases the expression levels of IL-6 and TNF-*α* genes and proteins in mouse peritoneal macrophages. To investigate how the mechanism increases the transcription of the pro-inflammatory cytokines, we measured the protein in TLR4 signaling pathways following the different stimulation of LPS. TLR4 is associated with two parallel downstream signaling pathways. The first pathway, known as the TLR4–TBK1 pathway, is MyD88-independent and involves the molecules TRAF3 and TBK1 [17, 36]. The second pathway, called the TLR4–TAK1 pathway, is MyD88-dependent and operates through the molecules TRAF6 and TAK1. These two pathways play crucial roles in transmitting signals downstream of TLR4 activation. Our results have shown that after stimulation of mouse peritoneal macrophages for 0.5, 1, and 2 hr by 1 ng/ml LPS, the levels of p-TAK1 and p-p38 in peritoneal macrophages of B6.∆sNASP.lpr mice were higher than those of B6.lpr mice only after stimulation for 2 hr. In the 10 ng/ml and 100 ng/ml LPS stimulation groups, the levels of p-TAK1, p-p65, and p-p38 in peritoneal macrophages of B6.∆NASP.lpr mice were significantly higher than those of B6.lpr mice at 0.5 hr after LPS stimulation. Additionally, the expression levels of p-TAK1, p-p65, p-JNK, and p-p38 protein in peritoneal macrophages of B6. ∆NASP mice were higher than those of B6.WT mice after stimulated with 1 ng/ml LPS for 2 hr. The levels of p-TAK1, p-p65, p-JNK, and p-p38 in peritoneal macrophages of B6.∆NASP mice were significantly higher than those of B6.WT mice at 0.5 hr after stimulation with 10 and 100 ng/ml LPS. These results indicated that the mutation in sNASP gene enhances the sensitivity of the TLR4 receptor in mouse peritoneal macrophages by the TLR4–TAK1 signaling pathway. Furthermore, compared with B6.∆sNASP.lpr, B6.WT and B6.∆sNASP mouse peritoneal macrophages, the expression levels of TBK1 and IRF3 phosphorylated proteins in TLR4–TBK1 signal pathway were not significantly different. This study suggested that mutation of sNASP gene has no significant effect on TLR4–TBK1 signaling pathway in mouse peritoneal macrophages.

## 5. Conclusion

A variant of sNASP enhances the sensitivity of the TLR4 to aggravate the signaling pathway and increases the release of inflammatory cytokines. Despite the current knowledge of sNASP, further investigations are needed to elucidate whether the mutant sNASP exerts similar effects on other TLRs and whether its involvement in histone acetylation modification and DNA methylation, which may contribute to the SLE progression [24, 37]. These additional investigations will provide further insights into the broader impact of the mutant sNASP and its role in modulating immune responses associated with SLE.

## Data Availability

The data used to support the findings of this study are included within the article.

## Supplementary Materials

Supplementary Materials Figure S1: sNASP mutation has no influence on IFN-*α* in B6.lpr mice.

## References

[1] Justiz Vaillant A. A., Goyal A., Varacallo M. (2022). *Systemic Lupus Erythematosus*.

[2] Shan J., Jin H., Xu Y. (2020). T cell metabolism: a new perspective on Th17/Treg cell imbalance in systemic lupus erythematosus. *Frontiers in Immunology*.

[3] Yin X., Kim K., Suetsugu H. (2021). Meta-analysis of 208370 East Asians identifies 113 susceptibility loci for systemic lupus erythematosus. *Annals of the Rheumatic Diseases*.

[4] Li W., Titov A. A., Morel L. (2017). An update on lupus animal models. *Current Opinion in Rheumatology*.

[5] Morel L., Rudofsky U. H., Longmate J. A., Schiffenbauer J., Wakeland E. K. (1994). Polygenic control of susceptibility to murine systemic lupus erythematosus. *Immunity*.

[6] Ju J., Xu J., Zhu Y., Fu X., Morel L., Xu Z. (2019). A variant of the histone-binding protein snasp contributes to mouse lupus. *Frontiers in Immunology*.

[7] Zhang J., Du X., Wang H. (2021). A variant of sNASP exacerbates lymphocyte subset disorder and nephritis in a spontaneous lupus model Sle1.Yaa mouse. *Mediators of Inflammation*.

[8] Bao H., Carraro M., Flury V. (2022). NASP maintains histone H3–H4 homeostasis through two distinct H3 binding modes. *Nucleic Acids Research*.

[9] Tirgar R., Davies J. P., Plate L., Nordman J. T., Copenhaver G. P. (2023). The histone chaperone NASP maintains H3–H4 reservoirs in the early Drosophila embryo. *PLoS Genetics*.

[10] Yang F.-M., Zuo Y., Zhou W. (2018). sNASP inhibits TLR signaling to regulate immune response in sepsis. *The Journal of Clinical Investigation*.

[11] Ma K., Li J., Wang X. (2018). TLR4^+^CXCR4^+^ plasma cells drive nephritis development in systemic lupus erythematosus. *Annals of the Rheumatic Diseases*.

[12] Brown G. J., Cañete P. F., Wang H. (2022). TLR7 gain-of-function genetic variation causes human lupus. *Nature*.

[13] Wen L., Zhang B., Wu X. (2023). Toll-like receptors 7 and 9 regulate the proliferation and differentiation of B cells in systemic lupus erythematosus. *Frontiers in Immunology*.

[14] Ray A., Dittel B. N. (2010). Isolation of mouse peritoneal cavity cells. *Journal of Visualized Experiments*.

[15] Alexopoulou L., Holt A. C., Medzhitov R., Flavell R. A. (2001). Recognition of double-stranded RNA and activation of NF-*κ*B by Toll-like receptor 3. *Nature*.

[16] Kenawy H. M., Marshall S. L., Rogot J., Lee A. J., Hung C. T., Chahine N. O. (2023). Blocking Toll-like receptor 4 mitigates static loading induced pro-inflammatory expression in intervertebral disc motion segments. *Journal of Biomechanics*.

[17] Häcker H., Redecke V., Blagoev B. (2006). Specificity in Toll-like receptor signalling through distinct effector functions of TRAF3 and TRAF6. *Nature*.

[18] Behzadi P., Garcia-Perdomo H. A., Karpinski T. M. (2021). Toll-like receptors: general molecular and structural biology. *Journal of Immunological Research*.

[19] Mande P., Zirak B., Ko W.-C. (2018). Fas ligand promotes an inducible TLR-dependent model of cutaneous lupus-like inflammation. *The Journal of Clinical Investigation*.

[20] Ronnblom L. (2016). The importance of the type I interferon system in autoimmunity. *Clinical and Experimental Rheumatology*.

[21] Tsukamoto H., Takeuchi S., Kubota K. (2018). Lipopolysaccharide (LPS)-binding protein stimulates CD14-dependent Toll-like receptor 4 internalization and LPS-induced TBK1-IKK*ε*-IRF3 axis activation. *Journal of Biological Chemistry*.

[22] Costa-Reis P., Sullivan K. E. (2013). Genetics and epigenetics of systemic lupus erythematosus. *Current Rheumatology Reports*.

[23] Morel L. (2010). Genetics of SLE: evidence from mouse models. *Nature Reviews Rheumatology*.

[24] Xu Z., Butfiloski E. J., Sobel E. S., Morel L. (2004). Mechanisms of peritoneal B-1a cells accumulation induced by murine lupus susceptibility locus *Sle*2. *The Journal of Immunology*.

[25] Xu Z., Xu J., Ju J., Morel L. (2017). A Skint6 allele potentially contributes to mouse lupus. *Genes and Immunity*.

[26] Ju J., Wang H., Lian M., Bao Y., Zhang Y., Xu Z. (2022). A murine Skint6 W168X allele contributes to autoimmune disease in a transgenic model. *Lupus*.

[27] Richardson R. T., Alekseev O. M., Grossman G. (2006). Nuclear autoantigenic sperm protein (NASP), a linker histone chaperone that is required for cell proliferation. *Journal of Biological Chemistry*.

[28] Apta-Smith M. J., Hernandez-Fernaud J. R., Bowman A. J. (2018). Evidence for the nuclear import of histones H3.1 and H4 as monomers. *The EMBO Journal*.

[29] Cook A. J. L., Gurard-Levin Z. A., Vassias I., Almouzni G. (2011). A specific function for the histone chaperone NASP to fine-tune a reservoir of soluble H3–H4 in the histone supply chain. *Molecular Cell*.

[30] Chen J.-Q., Szodoray P., Zeher M. (2016). Toll-like receptor pathways in autoimmune diseases. *Clinical Reviews in Allergy & Immunology*.

[31] Yang B., Huang X., Xu S. (2021). Decreased miR-4512 levels in monocytes and macrophages of individuals with systemic lupus erythematosus contribute to innate immune activation and neutrsophil NETosis by targeting TLR4 and CXCL2. *Frontiers in Immunology*.

[32] Achek A., Kwon H.-K., Patra M. C. (2020). A peptide derived from the core *β*-sheet region of TIRAP decoys TLR4 and reduces inflammatory and autoimmune symptoms in murine models. *EBioMedicine*.

[33] Aringer M. (2020). Inflammatory markers in systemic lupus erythematosus. *Journal of Autoimmunity*.

[34] Marczynski P., Meineck M., Xia N. (2021). Vascular inflammation and dysfunction in lupus-prone mice-IL-6 as mediator of disease initiation. *International Journal of Molecular Sciences*.

[35] Xiang S., Zhang J., Zhang M. (2022). Imbalance of helper T cell type 1, helper T cell type 2 and associated cytokines in patients with systemic lupus erythematosus: a meta-analysis. *Frontiers in Pharmacology*.

[36] Guven-Maiorov E., Keskin O., Gursoy A. (2015). The architecture of the TIR domain signalosome in the Toll-like receptor-4 signaling pathway. *Scientific Reports*.

[37] Hammond C. M., Strømme C. B., Huang H., Patel D. J., Groth A. (2017). Histone chaperone networks shaping chromatin function. *Nature Reviews Molecular Cell Biology*.

