# Supplementary material for: sNASP Mutation Aggravates to the TLR4-Mediated Inflammation in SLE by TAK1 Pathway
Source: J Immunol Res. 2023 Sep 20;2023:4877700. doi: 10.1155/2023/4877700 (PMC10533267; doi:10.1155/2023/4877700)
Supplement: Supplementary Materials — Figure S1: sNASP mutation has no influence on IFN-α in B6.lpr mice. [file 4877700.f1.docx]

Supplementary Data


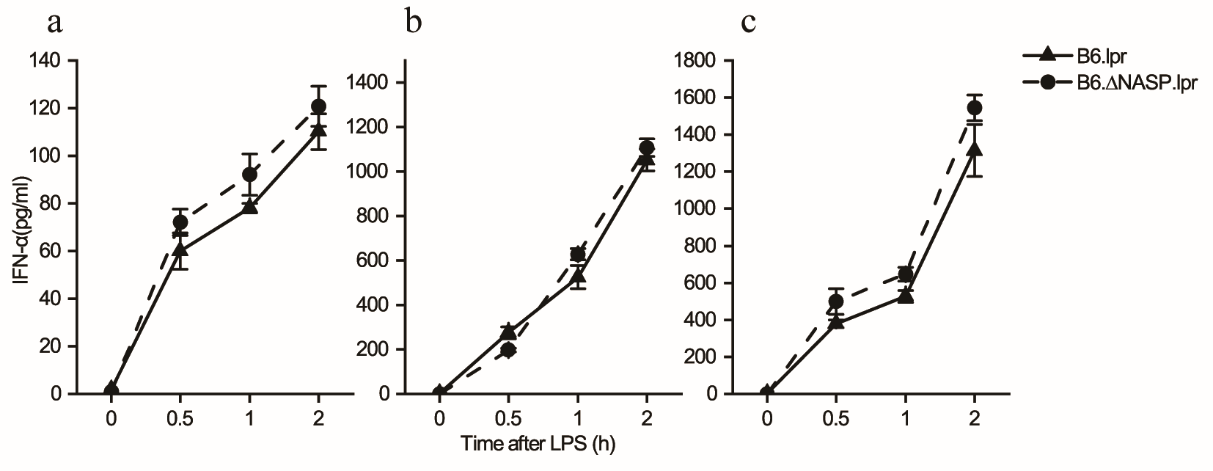


Figure S1. *sNASP* mutation has no influence on IFN-α in B6.lpr mice. The expression of protein (a-c) of IFN-α were measured by and ELISA in peritoneal macrophages, compared between B6.lpr and B6.∆sNASP.lpr mice. Following 0 h/ 0.5 h/ 1 h/ 2 h stimulation with (a) LPS 1 ng/ml, (b) LPS 10 ng/ml, and (c) LPS 100 ng/ml. Data was shown as means ± SEM (n=3) of one representative experiment. **P*<0.05, ***P*<0.01.
